# Targeting NPL4 via drug repositioning using disulfiram for the treatment of clear cell renal cell carcinoma

**DOI:** 10.1371/journal.pone.0236119

**Published:** 2020-07-15

**Authors:** Hirofumi Yoshino, Yasutoshi Yamada, Hideki Enokida, Yoichi Osako, Masafumi Tsuruda, Kazuki Kuroshima, Takashi Sakaguchi, Satoshi Sugita, Shuichi Tatarano, Masayuki Nakagawa

**Affiliations:** Department of Urology, Graduate School of Medical and Dental Sciences, Kagoshima University, Kagoshima, Japan; University of California San Francisco, UNITED STATES

## Abstract

The alcohol-abuse drug disulfiram has antitumor effects against diverse cancer types via inhibition of the ubiquitin-proteasome protein nuclear protein localization protein 4 (NPL4). However, the antitumor effects of NPL4 and disulfiram in clear cell renal cell carcinoma (ccRCC) are unclear. Here, we evaluated the therapeutic potential of targeting the ubiquitin-proteasome pathway using disulfiram and RNA interference and investigated the mechanisms underlying disulfiram in ccRCC. According to data from The Cancer Genome Atlas, *NPL4* mRNA expression was significantly upregulated in clinical ccRCC samples compared with that in normal kidney samples, and patients with high *NPL4* expression had poor overall survival compared with patients with low *NPL4* expression. Disulfiram and NPL4 siRNA inhibited ccRCC cell proliferation in vitro, and disulfiram inhibited ccRCC tumor growth in a xenograft model. Synergistic antiproliferative effects were observed for combination treatment with disulfiram and sunitinib in vitro and in vivo. In RCC cells from mice treated with disulfiram and/or sunitinib, several genes associated with serine biosynthesis and aldose reductase were downregulated in cells treated with disulfiram or sunitinib alone and further downregulated in cells treated with both disulfiram and sunitinib. These findings provided insights into the mechanisms of disulfiram and suggested novel therapeutic strategies for RCC treatment.

## Introduction

Renal cell carcinoma (RCC) is one of the most common human cancers of the kidney and the seventh most common tumor types [1]. At the time of diagnosis, nearly 30% of RCCs have already metastasized [2]. Surgical resection can effectively resolve clear cell RCC (ccRCC); however, 40% of patients suffer local recurrence or distinct metastasis following surgery [3]. To date, molecularly targeted therapeutics, such as multitargeted receptor tyrosine kinase (RTK) or mammalian target of rapamycin (mTOR) inhibitors, and anti-programmed death-1 (PD-1) antibodies have been widely used for patients with metastatic or recurrent RCC. Unfortunately, most patients treated with these drugs eventually suffer from progressive disease owing to intrinsic or acquired resistance [4,5], and the antitumor effects of these drugs have shown limited benefits. Therefore, the 5-year survival rate of patients with advanced-stage RCC is still less than 20%, even though survival rates have increased [6]. Accordingly, in order to improve outcomes in patients with RCC, it is necessary to identify new therapeutic approaches for the treatment of RCC.

Our recent studies showed that RCC has a diverse range of metabolites and that activation of serine biosynthesis can be targeted by inhibition of phosphoglycerate dehydrogenase (PHGDH) [7]. In addition, we also showed that bromodomain containing 4 (BRD4), a member of the bromodomain family proteins, can be targeted in RCC [8]. In both studies, in vivo and in vitro analyses showed antitumor effects in RCCs using PHGDH or BRD4 inhibitors, which exhibit mechanisms different from those of RTK and mTOR inhibitors or anti-PD-1 antibodies. Recently, the alcohol-abuse drug disulfiram has been shown to have antitumor effects against diverse cancer types via inhibition of nuclear protein localization protein 4 (NPL4), which is involved in the ubiquitin-proteasome pathway [9]. Although disulfiram is known to have antitumor effects, this was the first study showing the involvement of a mechanism associated with the ubiquitin-proteasome pathway. However, the potential therapeutic applications of targeting the ubiquitin-proteasome pathway using disulfiram in RCC have not been investigated. In addition, the ubiquitin-proteasome pathway is different from other pathways that are already being exploited in currently available treatments for ccRCC.

Accordingly, in this study, we investigated the anticancer efficacy of disulfiram in vitro and in vivo and examined the molecular mechanisms underlying disulfiram inhibition in RCC. First, we investigated the clinical relevance of NPL4 using data on ccRCC from The Cancer Genome Atlas (TCGA). In addition, we evaluated the therapeutic potential of disulfiram in vitro and in vivo using RCC cells. For further analyses, we performed combined treatment with disulfiram and sunitinib in vitro and in vivo in RCC cells because sunitinib, which mainly inhibits angiogenesis through suppression of vascular endothelial growth factor receptor (VEGFR), is clinically used as a first-line and second-line therapy for patients with advanced RCC. Moreover, we performed proteomic analyses of RCC cell fractions from mice treated with disulfiram and/or sunitinib to discover novel pathways underlying the effects of disulfiram in RCC cells.

## Materials and methods

### Cell culture

A human embryonic kidney cell line (HEK-293) was obtained from TaKaRa (Japan), human kidney cortex/proximal tubule epithelial cell line (HK2), and human RCC cell lines 786-o, A498, Caki1, and Caki2 were obtained from the American Type Culture Collection (ATCC, Manassas, VA, USA) in 2010. They were maintained in culture for no more than 30 continuous passages. ATCC authenticated cell lines with short tandem repeat profiling. All cell lines were tested and found negative for mycoplasma (e-Myco Mycoplasma PCR Detection Kit; iNtRON Biotechnology, Korea). Cells were incubated in RPMI 1640 medium (Thermo Fisher Scientific, Waltham, MA, USA) supplemented with 10% fetal bovine serum and maintained in a humidified incubator (5% CO_2_) at 37°C.

### Transfection with small interfering RNA (siRNA)

As described elsewhere [10], RCC cells were transfected with Lipofectamine RNAiMAX transfection reagent (Thermo Fisher Scientific) and Opti-MEM (Thermo Fisher Scientific) with 10 nM siRNA. NPL4 siRNA (SASI_ Hs02_00350914 and SASI_ Hs02_00350915; Sigma-Aldrich, St. Louis, MO, USA) and negative-control siRNA (D-001810-10; Thermo Fisher Scientific) were used in loss-of-function experiments.

### Cell proliferation, apoptosis, and caspase-3/7 activity assays

At 72 or 96 h after addition of siRNA, disulfiram (stock solution DMSO, Tocris Bioscience, Bristol, UK), or sunitinib (stock solution DMSO, Biorbyt, Cambridge, UK), cell proliferation was determined by XTT assays using a Cell Proliferation kit II (Roche Molecular Biochemicals, Mannheim, Germany) according to the manufacturer’s instructions. Cell apoptosis assays were carried out within 1 h by flow cytometry (CytoFLEX analyzer; Beckman Coulter, Brea, CA, USA) using a FITC Annexin V Apoptosis Detection Kit (BD Biosciences) according to the manufacturer’s recommendations. Cells were separated into viable cells, dead cells, and early and late apoptotic cells using CellQuest software (BD Biosciences, Bedford, MA, USA), and the percentage of total apoptotic cells from each sample was compared. Caspase-3/7 activity was measured using CellEvent caspase-3/7 Green Detection Reagent (Invitrogen, Carlsbad, CA, USA). Initially, RCC cell lines grown in 96-well plates were treated with control, 1 μM disulfiram, 10 μM sunitinib, or a combination of 1 μM disulfiram and 10 μM sunitinib. After 48 h, 5 μM caspase-3/7 reagent was added to each well and incubated for 30 min. Fluorescence was then measured and recorded for each well. For the analysis, the measurements were normalized to the cell proliferation results obtained by XTT assays performed at the same time (48 h). Experiments were carried out in triplicate. In this study, we adopted sunitinib concentration as 10 μM according to previous study [7], in which 5 μM sunitinib showed inhibitory effect of 786-o cell proliferation by around 30%, whereas 10 μM sunitinib showed it by 80%.

### Cell migration and invasion assays

Cell migration activity was evaluated with wound healing assays. Cell invasion assays were performed using modified Boyden chambers consisting of Transwell precoated Matrigel membrane filter inserts with 8-μm pores in 24-well tissue culture plates (BD Biosciences). The experimental procedures were performed as described in our previous study [11].

### Immunoassays

Western blot analysis was carried out as previously described [12] with diluted (1:1,000) anti-NPL4 antibodies (Novus bio, CO, USA), anti-AKR1B1 antibodies (GTX113381; Gene Tex, Inc., Irvine CA, USA), anti-PSAT1 antibodies (GTX633623; Gene Tex), and anti-b-actin antibodies (bs-0061R; Bioss). For immunofluorescence analyses, nuclei were stained with DAPI (1 mg/mL; Kirkegaard and Perry Laboratories), and slides were mounted in Fluoromount (Diagnostic Biosystems). Anti-NPL4 antibodies were used as the primary antibody at a dilution of 1:100, and binding was visualized using secondary antibodies conjugated to Alexa Fluor 488 (ab150077; Abcam).

### In vivo tumor xenograft model

A mixture containing 100 uL 786-o cells (2 × 10^6^ cells) and 100 uL Matrigel Matrix (Corning, NY, USA) was injected subcutaneously into the flanks of female nude mice (BALB/c nu/nu, 6–8 weeks old). Mice were anesthetized with 1% to 3% isoflurane in 2 L/min oxygen during cell injection. Mice were separated into four groups: vehicle, disulfiram (200 mg/kg, administered by oral gavage, 5 times a week), sunitinib (40 mg/kg, administered by oral gavage, 5 times a week), or the combination of disulfiram and sunitinib. The dose was adjusted according to the weight of each mouse. Mice condition was monitored 5 times a week, and all mice were euthanized by cervical dislocation when the xenografts were harvested. All animal experiments were approved by the animal care review board of Kagoshima University.

### Proteomics analyses with tumor sections obtained from the xenograft model

In vitro proteome-assisted multiple reaction monitoring for protein absolute quantification analysis was performed by using tumor fractions from our in vivo study at Kyusyu University in Japan [13]. In our proteomic analysis, we focused on 342 metabolic enzymes, excluding NPL4. We applied the analysis to search for unknown effects of disulfiram on metabolic enzymes.

### Overall survival analysis of a ccRCC cohort (KIRC) from TCGA

TCGA cohort data for 72 normal kidneys and 534 patients with ccRCC (KIRC) were used to determine the relationships between *NPLOC4* (*NPL4*) expression in normal tissues and in ccRCCs and between *NPL4* expression in ccRCC and overall survival. Normalized RNA-seq by expectation-maximization values from RNA-seq expression data were used for gene expression quantification [14]. Full sequencing information and clinical information were acquired from cBioPortal (http://www.cbioportal.org/public-portal/) and TCGA database (https://tcga-data.nci.nih.gov/tcga/) [15–17].

### Statistical analysis

Data are presented as means ± standard deviations of at least 3 independent experiments. Relationships between two or three variables and numerical values were analyzed using Mann-Whitney *U* tests or Bonferroni-adjusted Mann-Whitney *U* tests. Overall survival in patients with ccRCC from TCGA cohort was evaluated by the Kaplan-Meier method. Patients were numerically divided equally into two groups according to *NPL4* expression, and differences between the two groups were evaluated by log-rank tests. We used Expert Stat View software, version 5.0 (Cary, NC, USA) for these analyses. When *P* < 0.05, the data were accepted as showing a statistically significant difference.

## Results

### Clinical significance of NPL4 expression in ccRCC

**Fig 1:**
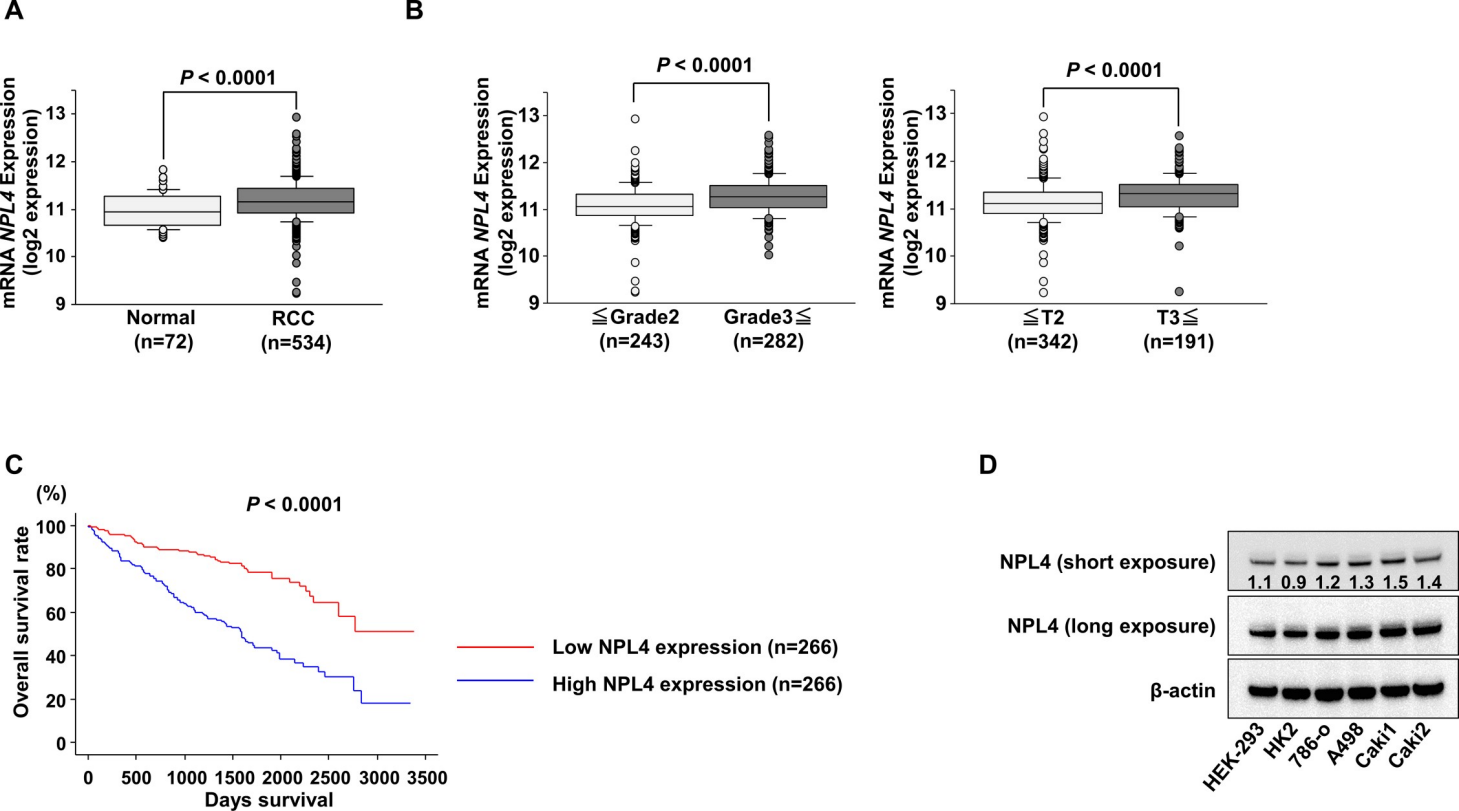
Analysis of TCGA kidney clear cell carcinoma (KIRC) datasets. (A) Expression levels of *NPL4* in normal kidney tissues and ccRCC specimens were determined using KIRC data. (B) In the ccRCC cohort of TCGA, the expression levels of *NPL4* were significantly increased in pathological G3 and G4 cases (left) and pathological T3 and T4 categories (right). (C) Kaplan-Meier survival curves of overall survival rates based on *NPL4* expression in 532 patients with ccRCC. *P* values were calculated using log-rank tests. (D) NPL4 protein expression levels in HEK-293, HK2, and RCC cell lines were determined by western blotting.

The expression levels of *NPL4* were significantly higher in ccRCCs than in normal kidneys in data obtained from TCGA database (*P* < 0.0001; Fig 1A). In addition, the expression level of *NPL4* was significantly increased in grade 3 and 4 cases compared with that in grade 1 and 2 cases and in pathological T3 and 4 cases compared with that in T1 and 2 cases (Fig 1B). We then evaluated the correlation between *NPL4* expression level and overall survival in patients with ccRCC. The cohort was numerically divided equally into two groups based on *NPL4* expression. We found that the low *NPL4* expression group (n = 266) had better median overall survival compared with the high *NPL4* expression group (n = 266; *P* < 0.0001; Fig 1C). Furthermore, western blot analyses demonstrated that NPL4 protein expressions in several ccRCC cells were elevated in comparison with the levels in human kidney HEK-293 and HK2 cells (Fig 1D).

### Effects of NPL4 inhibition by siRNA or disulfiram in RCC cells

**Fig 2:**
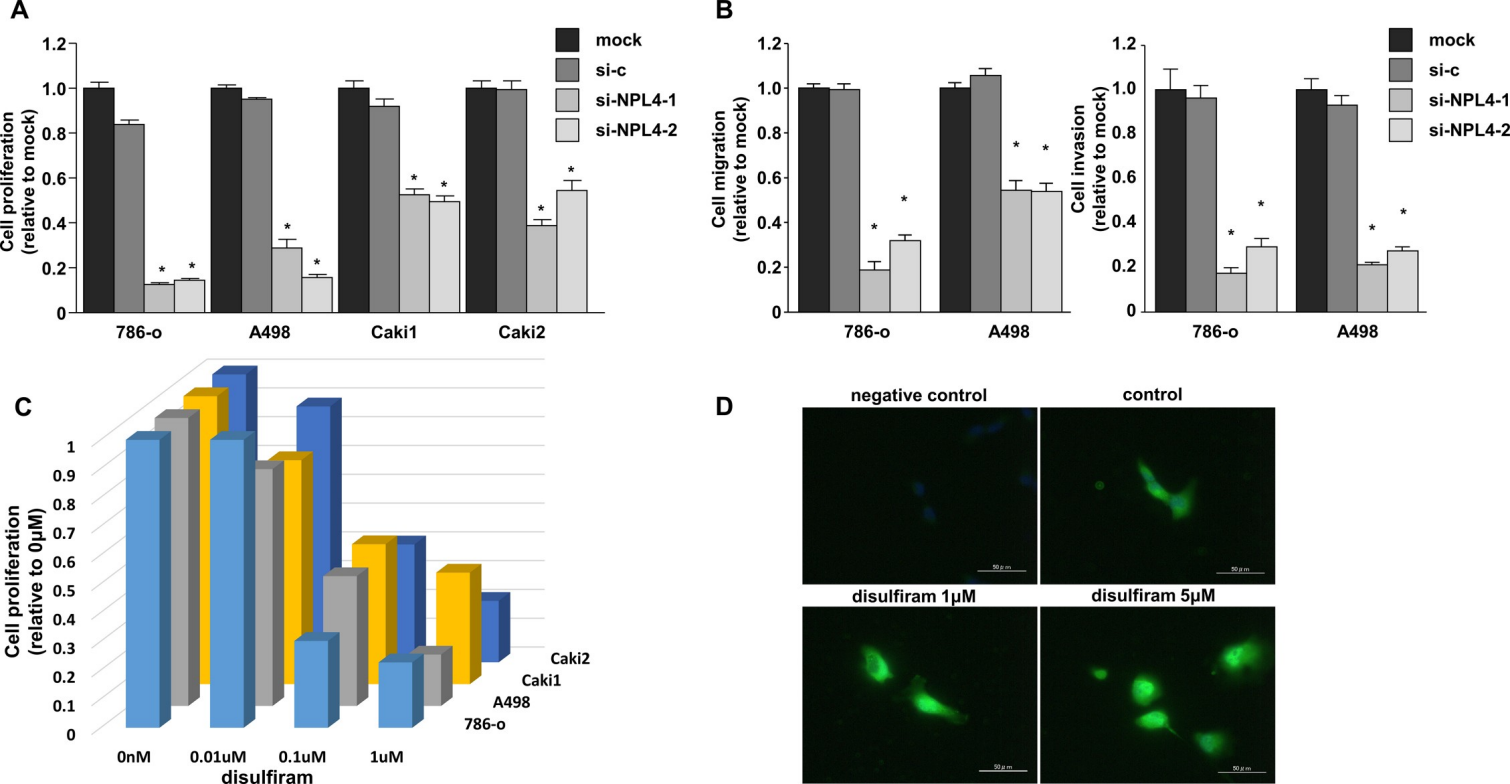
Effects of siNPL4 transfection and disulfiram on RCC cell lines. (A) Cell proliferation was determined by XTT assays at 96 h after transfection with siNPL4 (A; *, *P <* 0.0001). (B) Wound healing (left) and matrigel invasion (right) assays after transfection with siNPL4 (*P < 0.0001). (C) Cell proliferation was determined by XTT assays at 96 h after treatment with disulfiram. (D) Representative image of immunofluorescence analysis in cells with or without disulfiram. Anti-NPL4 antibody was not used in negative control.

Cell proliferation was inhibited by transfection of RCC cells with si*NPL4* compared with that in mock or siControl transfection (*P* < 0.0001; Fig 2A). Cell migration and invasion were also inhibited by transfection of RCC cells with si*NPL4* compared with that in mock or siControl transfection (*P* < 0.0001; Fig 2B). Furthermore, cell proliferation was inhibited by disulfiram in a concentration-dependent manner (Fig 2C). Immunofluorescence analyses showed NPL4 aggregation with its increased intensity in 786-o cells after disulfiram treatment (Fig 2D).

### Effects of disulfiram and sunitinib in RCC cells

**Fig 3:**
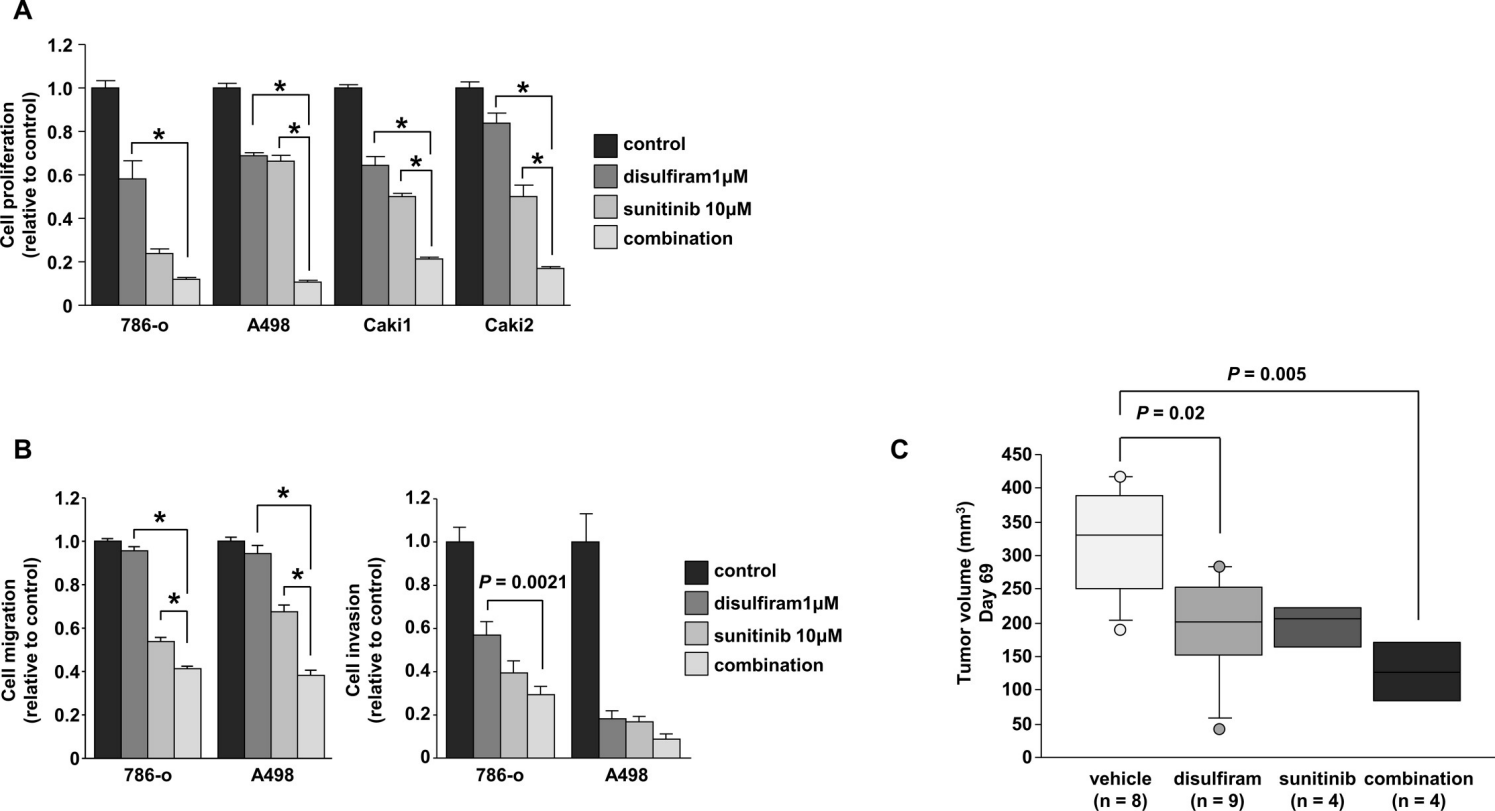
Synergistic effects of the combination of disulfiram and sunitinib in RCC. (A) In vitro cell proliferation assays following treatment with disulfiram, sunitinib, or the combination of disulfiram and sunitinib after 72 h (*P < 0.001). (B) Wound healing and matrigel invasion assays after treatment with disulfiram. (*P < 0.0001). (C) In vivo cell proliferation assays; Tumor volumes were calculated in nude mice 69 days after subcutaneous injection of 786-o cells treated with vehicle, disulfiram, sunitinib, or the combination of disulfiram and sunitinib. Four groups were examined: vehicle, disulfiram (200 mg/kg, 5 times a week), sunitinib (40 mg/kg, 5 times a week), and a combination of disulfiram and sunitinib (n = 8 for vehicle, n = 9 for disulfiram group, n = 4 for the sunitinib and combination groups).

Next, we examined the effects of combining treatments with disulfiram and sunitinib. Cell proliferation data showed significant synergistic effects on tumor suppression compared with that achieved with the individual agents (*P* < 0.001; Fig 3A). Cell migration assays also showed significant synergistic effects on tumor migration compared with that achieved with the individual agents (*P* < 0.0001; Fig 3B, left). In contrast, there was a tendency for synergism in assays of cell invasion, although the differences were not significant aside from that between disulfiram and combination in 786-o cells (P = 0.0021; Fig 3B, right). We also performed xenograft assays with disulfiram plus sunitinib. The results showed that tumor growth was significantly suppressed in mice treated with disulfiram compared with that in vehicle-treated mice (*P* = 0.02). In addition, combined treatment using disulfiram and sunitinib showed synergistic effects on tumor suppression compared with vehicle-treated mice (*P* = 0.005), although the differences in tumor volume suppression between the combined group and single agent groups were not significant (Fig 3C). The relationships among the treatment groups at each time point were analyzed using Bonferroni-adjusted Mann-Whitney U tests to adjust for multiplicity.

### Disulfiram induced apoptosis through caspase-3/7 activation in RCC cell lines

**Fig 4:**
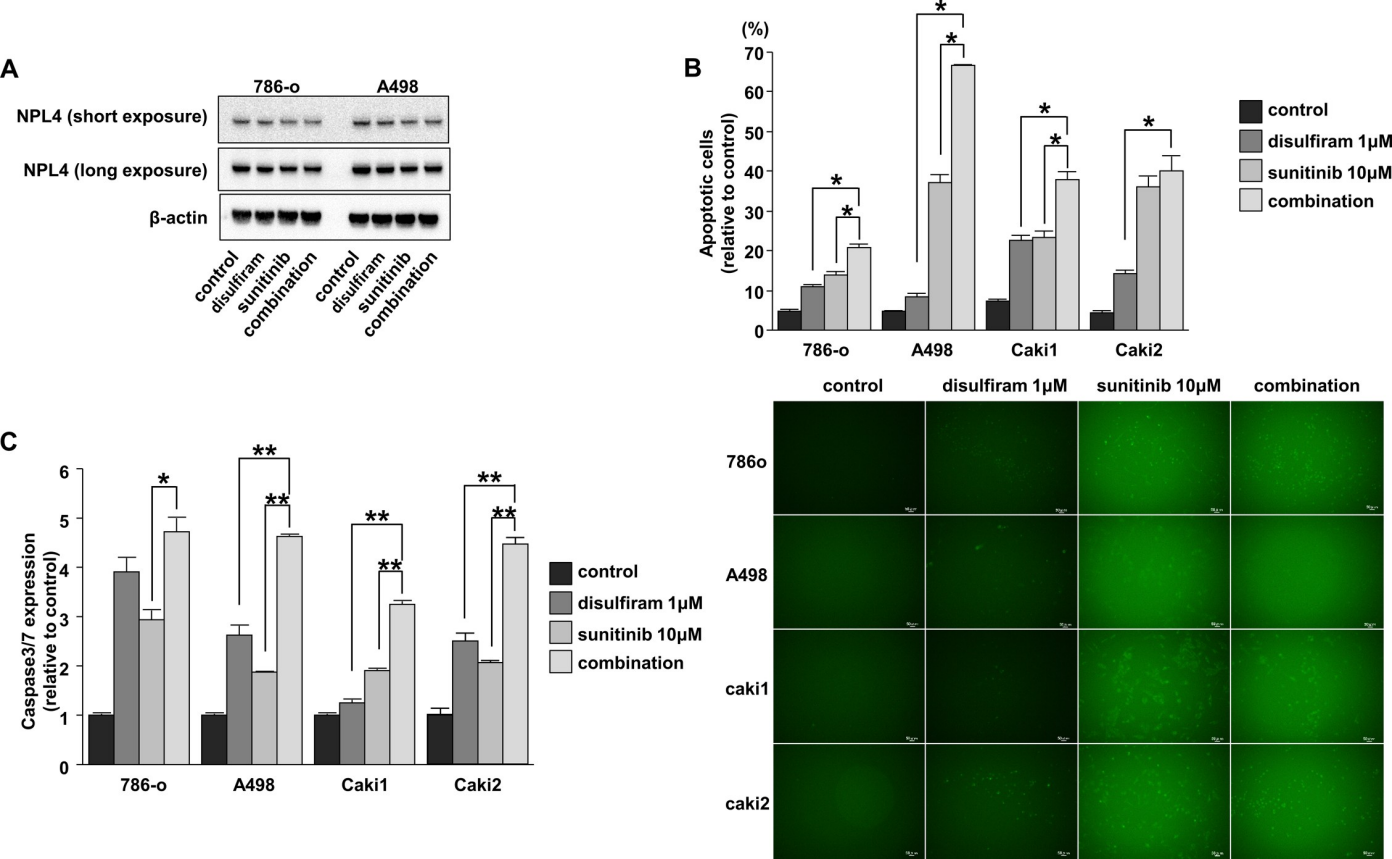
Effects of disulfiram and sunitinib on apoptosis and detection of caspase-3/7 activation in RCC cell lines. (A) NPL4 protein expression levels in RCC cell lines treated with control, disulfiram, sunitinib, or the combination of disulfiram and sunitinib for 48 h were determined by western blotting analyses. (B) Apoptosis assays were performed using flow cytometry at 72 h after drug treatment (*, *P* < 0.0001). (C) Caspase-3/7 activity was measured by determining fluorescence intensity in RCC cells treated with control, disulfiram, sunitinib, or the combination of disulfiram and sunitinib for 48 h (*, *P* < 0.001; **, *P* < 0.0001; left). Representative images of caspase-3/7 activity in RCC cell lines (right).

To elucidate the mechanisms mediating the synergistic effects of disulfiram and sunitinib, we evaluated NPL4 expression levels in cells treated with control, disulfiram, sunitinib, or the combination of disulfiram and sunitinib. Western blot analysis showed slight decrease of NPL4 in sunitinib or the combination compared with control or disulfiram (Fig 4A). Apoptosis was detected using flow cytometry after 72 h treatment with disulfiram. The apoptotic cell fractions were significantly greater after combined treatment with disulfiram or sunitinib in 786-o, A498, and Caki1 cells (Fig 4B). In Caki2 cells, there were no significant differences between sunitinib and the combination treatment, whereas significantly increased apoptotic cell fractions were observed after combination treatment compared with disulfiram treatment alone (Fig 4B). Caspase3/7 activity assays showed that fluorescent intensity was significantly increased after combination treatment compared with that after disulfiram treatment alone in A498, Caki1, and Caki2 cells and compared with that after sunitinib treatment alone in 786-o, A498, Caki1, and Caki2 cells (Fig 4C).

### Proteomic analyses of RCC cell fractions from mice treated with disulfiram and/or sunitinib

**Fig 5:**
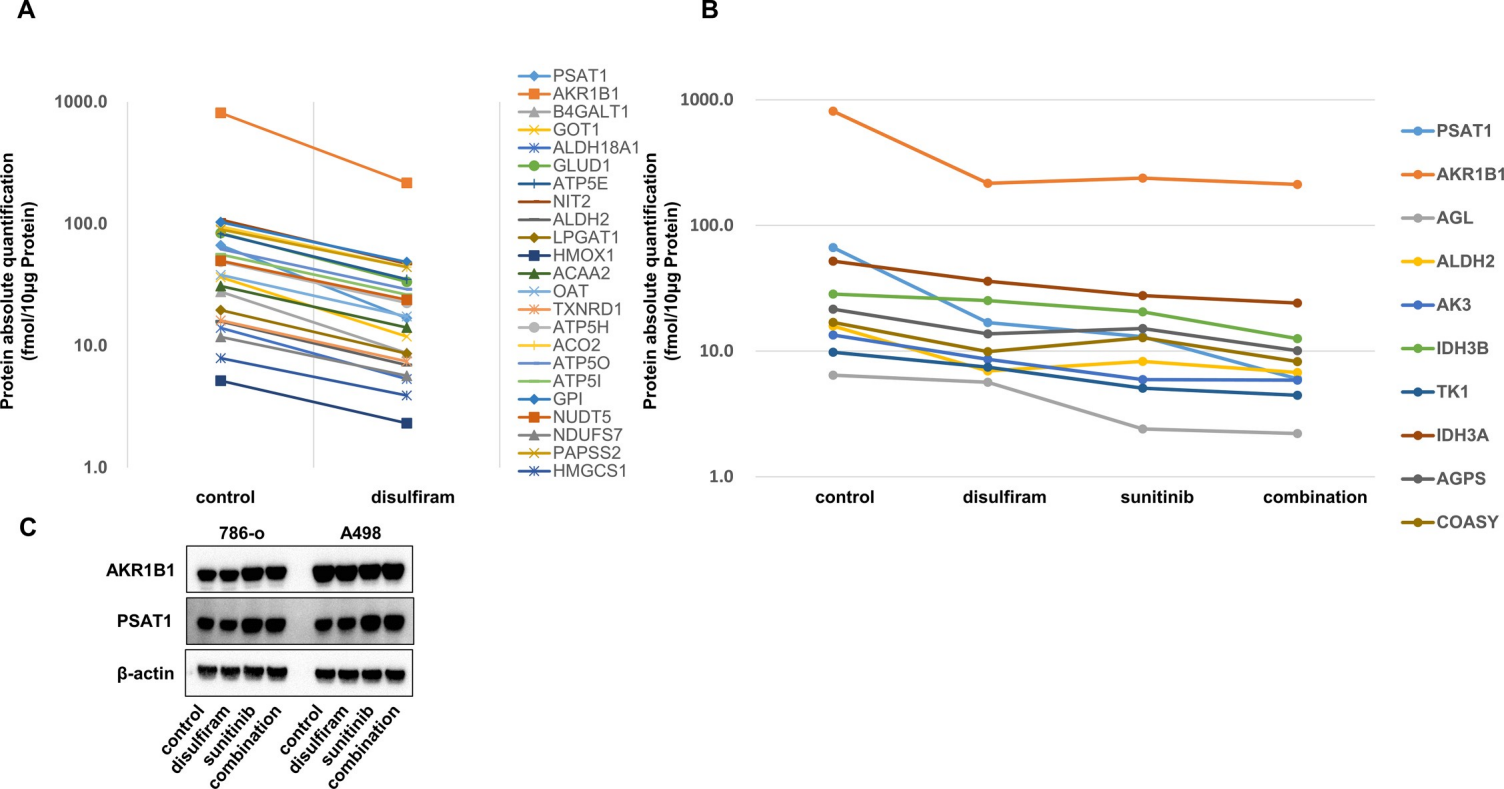
Proteomic analyses of RCC cell fractions from mice treated with disulfiram and/or sunitinib. Absolute quantification of proteins downregulated by disulfiram (A) or combination treatment with disulfiram and sunitinib (B). (C) AKR1B1 and PSAT1 protein expression levels in RCC cell lines treated with control, disulfiram, sunitinib, or the combination of disulfiram and sunitinib for 48 h were determined by western blotting analyses.

Quantitative proteomics analyses were performed by using tumor fractions from mice in Fig 3C to search for unknown effects of disulfiram other than NPL4. The results showed that there were several proteins for which the absolute quantity was decreased by at least 50% (Fig 5A). Quantitative proteomics analyses also showed that proteins were further downregulated in cells treated with the combination of both disulfiram and sunitinib compared with that in cells treated with disulfiram or sunitinib alone (Fig 5B). Proteomics analyses showed that disulfiram decreased the expression of aldo-keto reductase family 1, member B1 (AKR1B1) and phosphoserine aminotransferase 1 (PSAT1) by more than 70%, and several genes were also decreased not only by disulfiram but also by sunitinib or the combination. We then examined the expression levels of AKR1B1 and PSAT1 by using RCC cells in vitro. However, AKR1B1 and PSAT1 levels were not decreased in cells treated with disulfiram (Fig 5C). We assumed that the reason for its discrepancy may have been related to differences among samples; tumor fractions were used in the proteomics analyses, whereas cells treated with disulfiram for only 72 h in vitro were used in western blot analyses.

## Discussion

NPL4, one of the most important cofactors of valosin-containing protein (p97) together with Ufd1, is involved in many p97-dependent processes, including endoplasmic reticulum-associated protein degradation (ERAD), by segregating polyubiquitinated proteins from complexes or membranes [18]. Because cancer cells are characterized by high protein synthesis rates and rapid cell cycle progression, these cell rely heavily on ERAD pathways [19]. Moreover, several studies have shown that elevated expression of p97 is associated with poor prognosis in prostate cancer, esophageal cancer, pancreatic ductal adenocarcinoma, and non-small cell lung carcinoma [20–23]. In addition, increased p97 expression is associated with lymph node metastasis in pancreatic ductal adenocarcinoma and with disease recurrence in follicular thyroid cancer [22,24]. Because of the strong association between p97 expression and cancer prognosis, p97 inhibitors have also been shown to have inhibitory effects against various cancers. For example, Anderson et al. showed that the p97 inhibitor CB-5083 exerted antitumor activity through induction of apoptosis in several hematological and solid tumor models [19]. Furthermore, antitumor activity of CB-5083 was observed in xenograft models of colon cancer, non-small cell lung cancer, plasmacytoma, and colorectal cancer patient-derived xenograft models. Because of its promising anticancer activity, CB-5083 has been evaluated in phase I clinical trials for the treatment of patients with lymphoid hematologic malignancies [25]. However, these phase I clinical trials failed owing to unexpected off-target effects, necessitating further improvement of the inhibitor.

In contrast to p97, the roles of NPL4 in cancers have not been thoroughly evaluated. Skrott et al. reported that dysfunction of the p97 pathway owing to NPL4 aggregate formation in response to disulfiram causes cell death through inhibition of protein degradation with its increased intensity [9,26]. In this paper, NPL4 aggregation with its increased intensity was also indicated in 786-o cells after disulfiram treatment. Moreover, Lu et al. showed that NPL4 was upregulated in bladder cancer tissue and was correlated with poor prognosis; NPL4 knockdown was found to decrease bladder cancer cell proliferation by reduction of cyclin D1 expression [27]. Because NPL4 is the most important cofactor of p97, NPL4, which can be targeted by disulfiram, is also a key molecule for the treatment of cancers.

Disulfiram has synergistic antitumor effects with other drugs, including copper, doxorubicin, and temozolomide, in several cancers [28–30]. Interestingly, Ketola et al. indicated that sunitinib acted synergistically with disulfiram to induce apoptosis in prostate cancer cells [31]. Thus, our finding that disulfiram plus sunitinib treatment had additive antitumor effects through induction of apoptosis was plausible. However, the effects of disulfiram and sunitinib on cell proliferation and apoptosis differed in this study, particularly in A498 cells. We then postulated that the different mutational status of each cell line, as indicated in CCLE (https://portals.broadinstitute.org/ccle), may have contributed to the differences in cell proliferation and apoptosis. We anticipate that the combination of disulfiram and sunitinib may enable reduction of the dose of sunitinib, thereby avoiding severe adverse events and toxicity-related death. In addition, the benefits of combination therapy with disulfiram can also be applied to other drugs, such as pazopanib and axitinib, because these drugs are also clinically used to block tumor angiogenesis through VEGFR inhibition, similar to sunitinib for the treatment of RCC. Interestingly, Zhou et al. demonstrated that combination therapy with disulfiram/Cu^2+^ and an anti-PD-1 antibody showed much better antitumor efficacy than monotherapy via PD-L1 stabilization in hepatocellular carcinoma [32]. Because anti-PD-1 antibody treatment is already used for patients with RCC exhibiting recurrence or metastasis and because combination therapies, such as anti-PD-1 antibody plus anti-CTLA4 antibody treatment or anti-PD-1 antibody plus axitinib treatment, have also been applied clinically in the treatment of RCC [33], combination therapy with disulfiram may be a promising approach. Clinical trials with these combination therapies are needed to improve cancer treatment options in the near future.

In order to investigate the NPL4-independent mechanisms of action of disulfiram, proteomics analyses were performed using tumor fractions from our in vivo study. The results showed that disulfiram decreased the expression of PSAT1 and AKR1B1 by more than 70%. PSAT1 is an enzyme that functions downstream of PHGDH in the serine biosynthetic pathway [34]. The serine biosynthetic pathway is essential for the maintenance of rapid, sustained, uncontrolled cell proliferation in cancer cells owing to the production of one-carbon units that contribute to de novo synthesis of purines and pyridines [35]. Interestingly, disulfiram inhibits PHGDH [36,37]; although PHGDH was not detected in this proteomics analysis, the serine biosynthetic pathway can be inhibited by disulfiram through PSAT1 inhibition. The aldo-keto reductase AKR1B1 is associated with diabetes mellitus and is broadly overexpressed in human cancers; AKR1B1 overexpression is related to shortened patient survival in acute myelogenous leukemia and multiple myelomas [38]. Moreover, AKR1B1 expression is significantly increased in RCC compared with that in corresponding normal kidney tissues [38]. Although it is unclear whether AKR1B1 expression is a biomarker for cancer or a potential target for cancer treatment, several studies have shown that AKR proteins play central roles in the cellular response to osmotic, electrophilic, and oxidative stress [39,40], suggesting potential applications in cancer therapy. Because we did not fully investigate the mechanisms associated with PSAT1 and AKR1B1 in this study, further investigations are necessary to improve our understanding of the mechanisms underlying the antitumor effects of disulfiram.

In conclusion, disulfiram showed excellent antitumor effects against RCC cells in vitro and vivo. In addition, additive antitumor effects were observed in combination treatment with disulfiram and sunitinib in vitro and vivo. Furthermore, our findings indicated that the antitumor effects observed in this study may be caused by downregulation of serine biosynthesis and aldose reductase. To the best of our knowledge, this is the first paper indicating the potential therapeutic applications of disulfiram in the treatment of RCC.

## Supporting information

S1 Checklist (PDF)

S1 Data (XLSX)

S1 File (ZIP)
