# Supplementary figures and images for: Targeting NPL4 via drug repositioning using disulfiram for the treatment of clear cell renal cell carcinoma
Source: PLoS One. 2020 Jul 15;15(7):e0236119. doi: 10.1371/journal.pone.0236119 (PMC7363112; doi:10.1371/journal.pone.0236119)

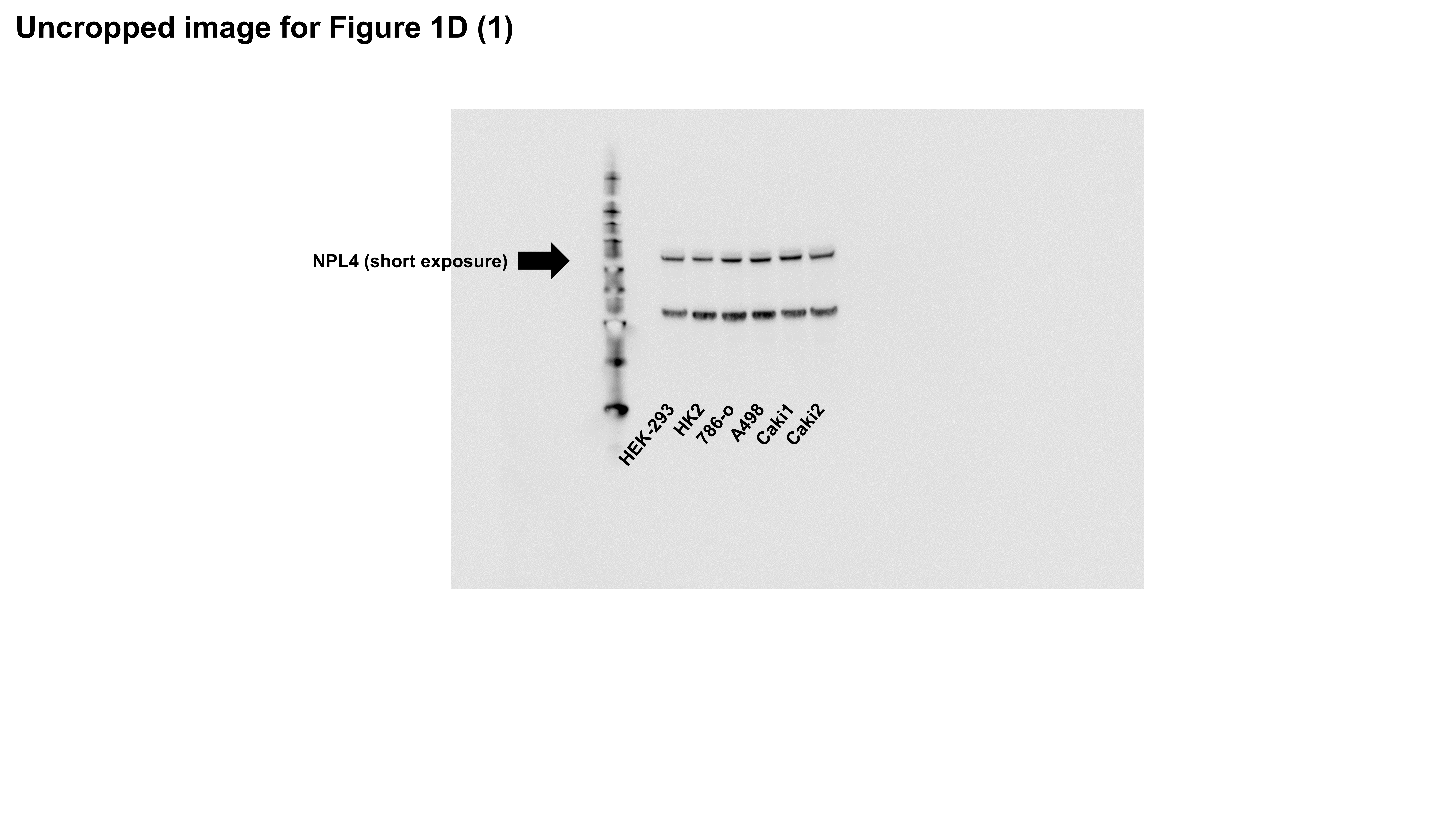

Supplement: S1 File — (ZIP) [file pone.0236119.s003.zip › S1 Fig..TIF]

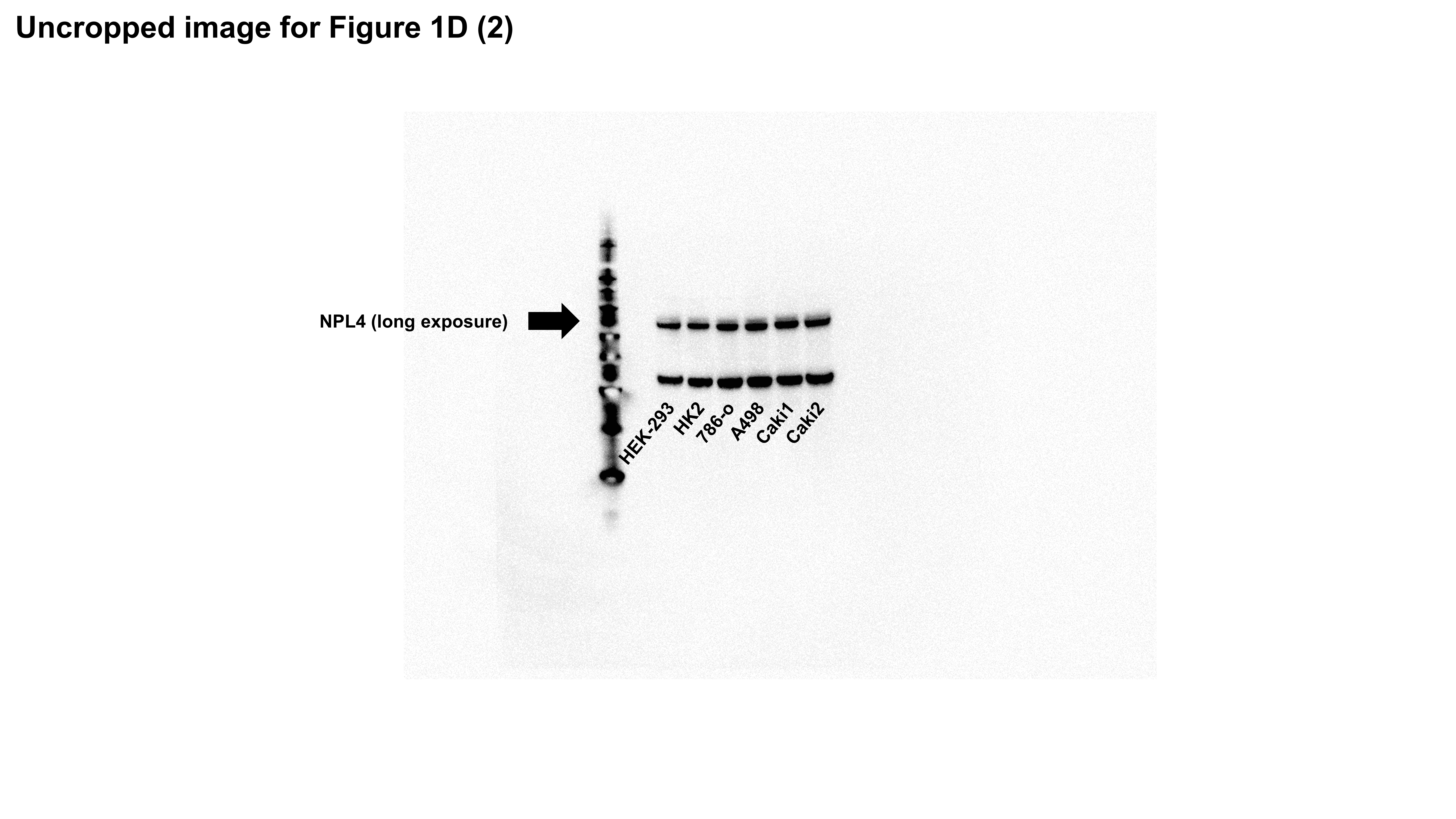

Supplement: S1 File — (ZIP) [file pone.0236119.s003.zip › S2 Fig..TIF]

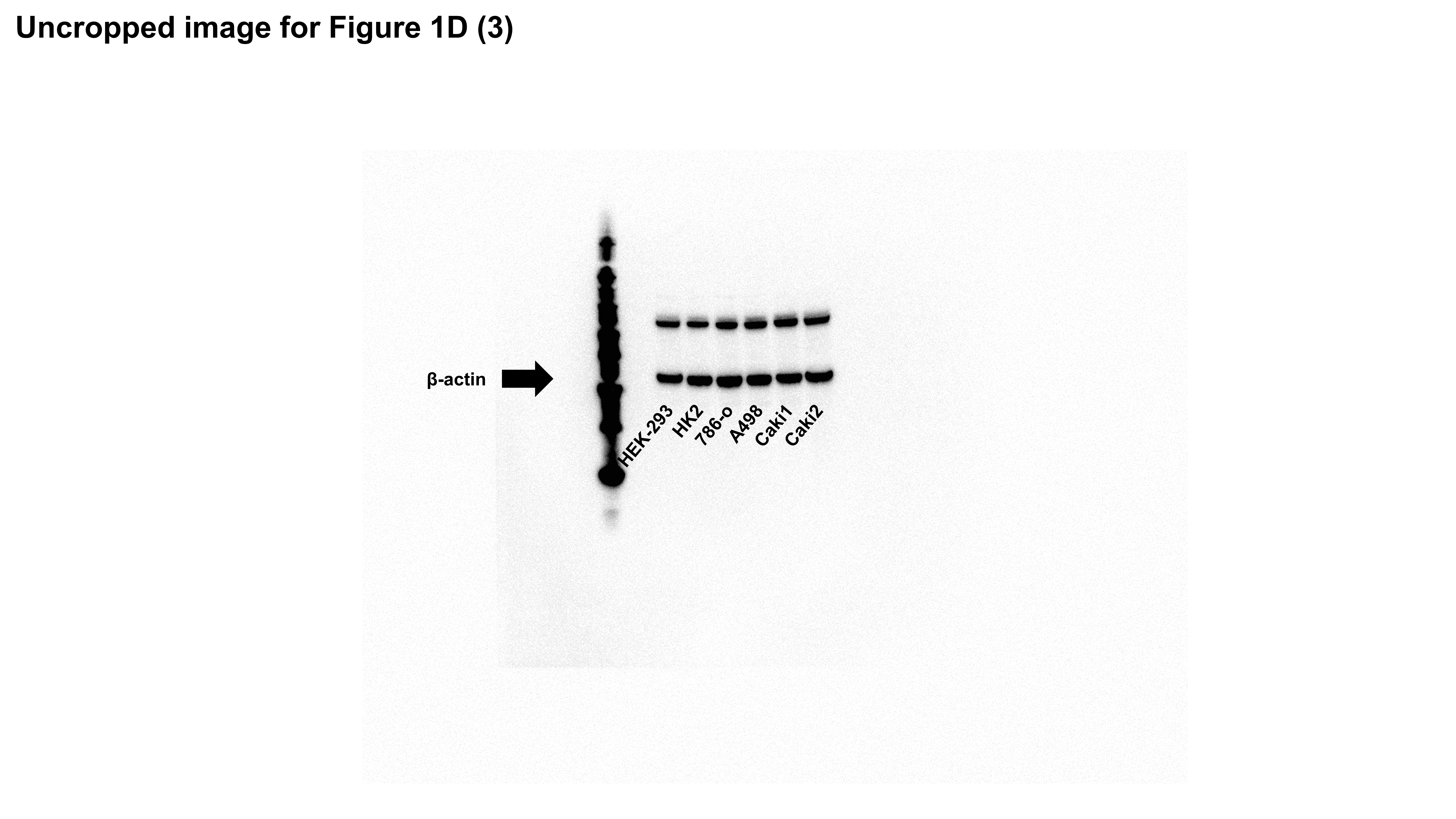

Supplement: S1 File — (ZIP) [file pone.0236119.s003.zip › S3 Fig..TIF]

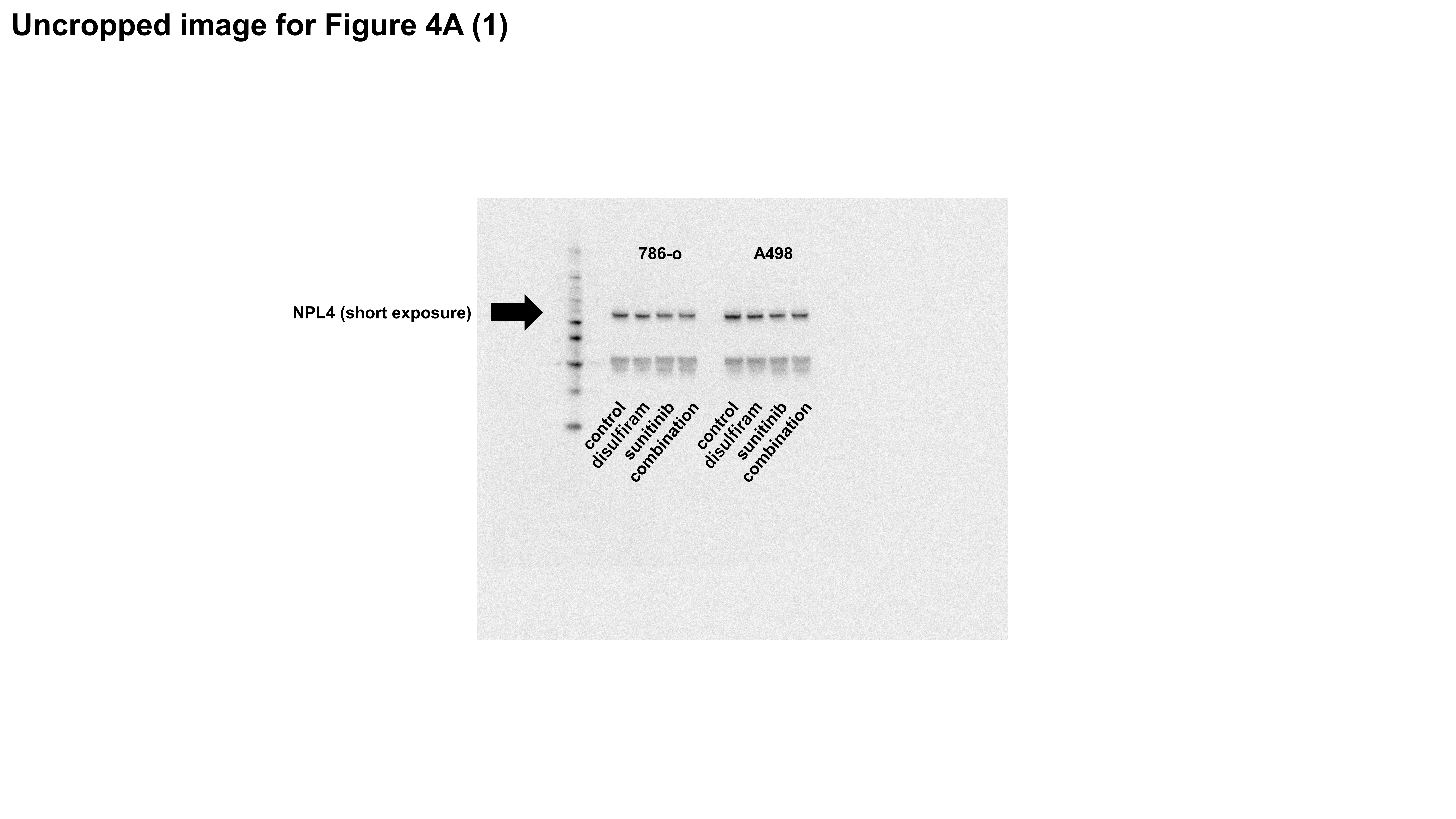

Supplement: S1 File — (ZIP) [file pone.0236119.s003.zip › S4 Fig..TIF]

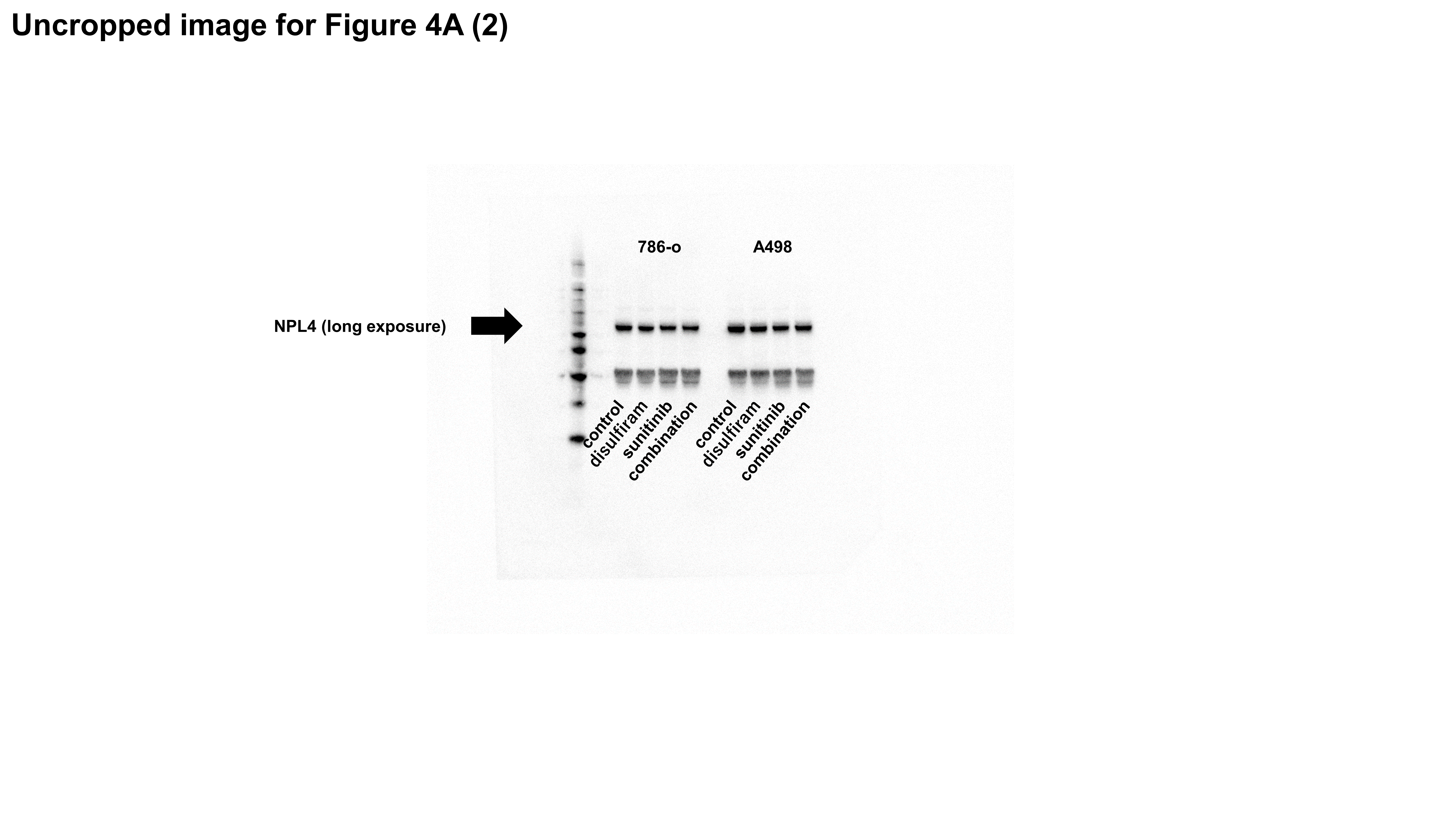

Supplement: S1 File — (ZIP) [file pone.0236119.s003.zip › S5 Fig..TIF]

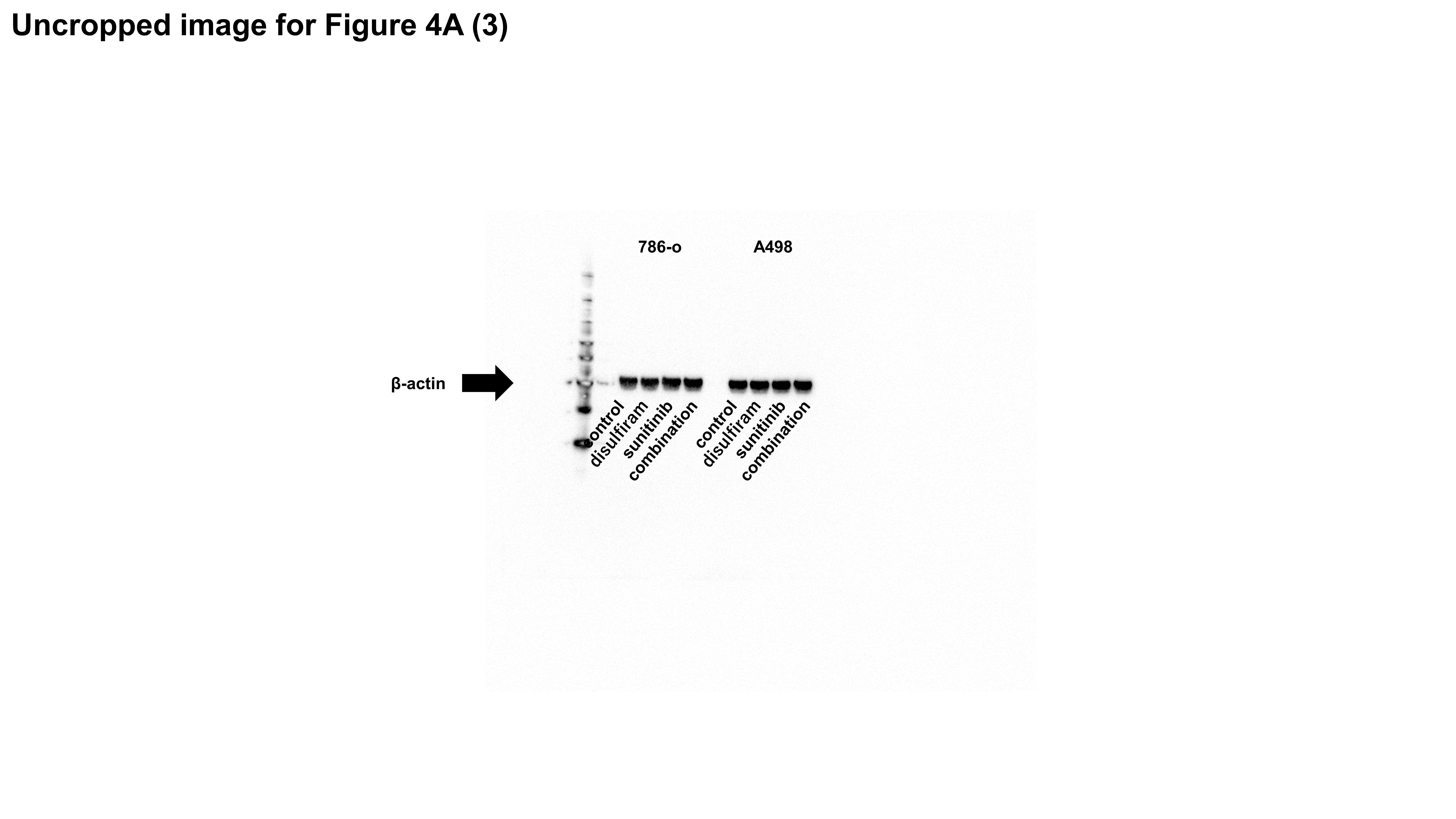

Supplement: S1 File — (ZIP) [file pone.0236119.s003.zip › S6 Fig..TIF]

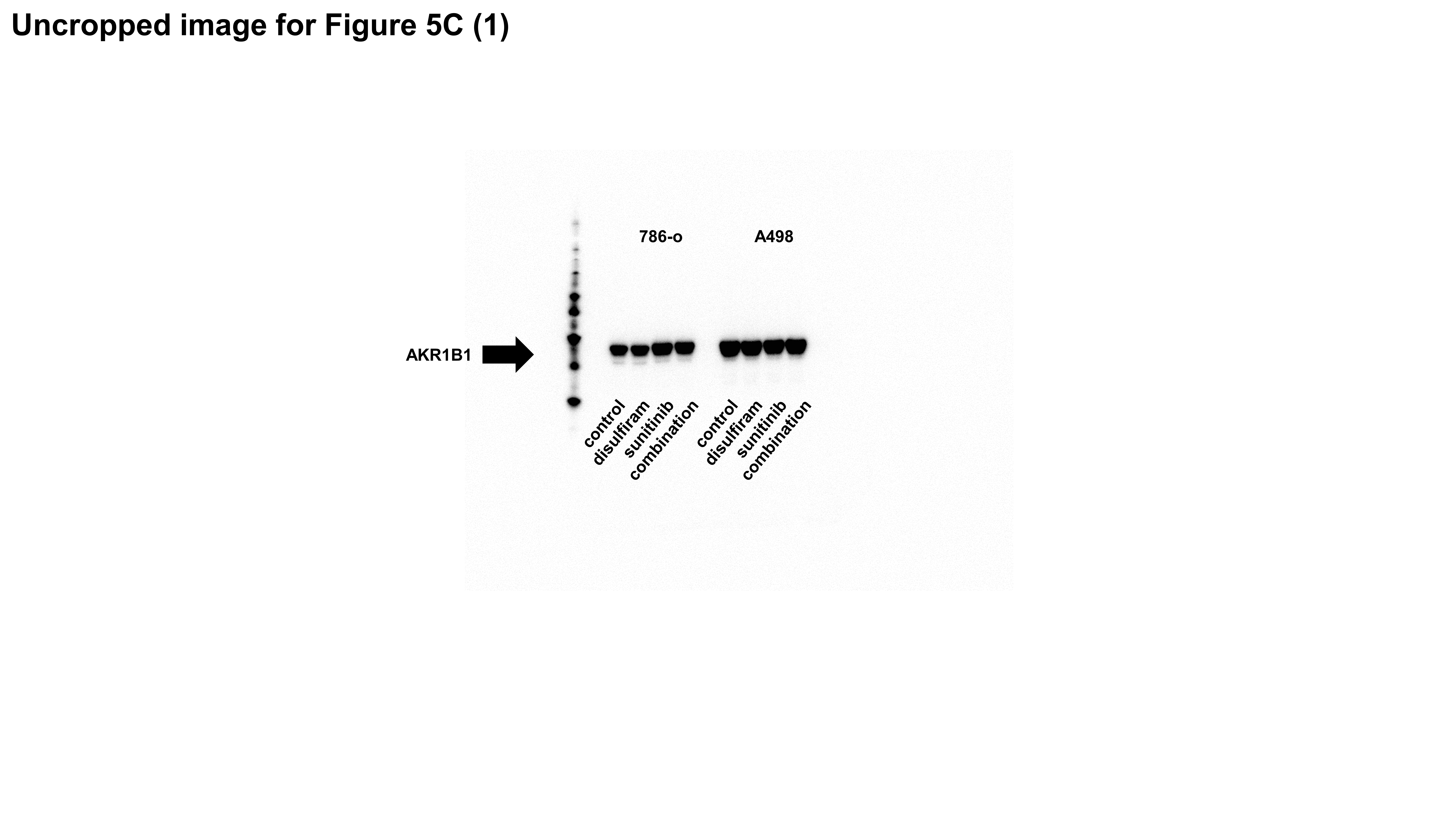

Supplement: S1 File — (ZIP) [file pone.0236119.s003.zip › S7 Fig..TIF]

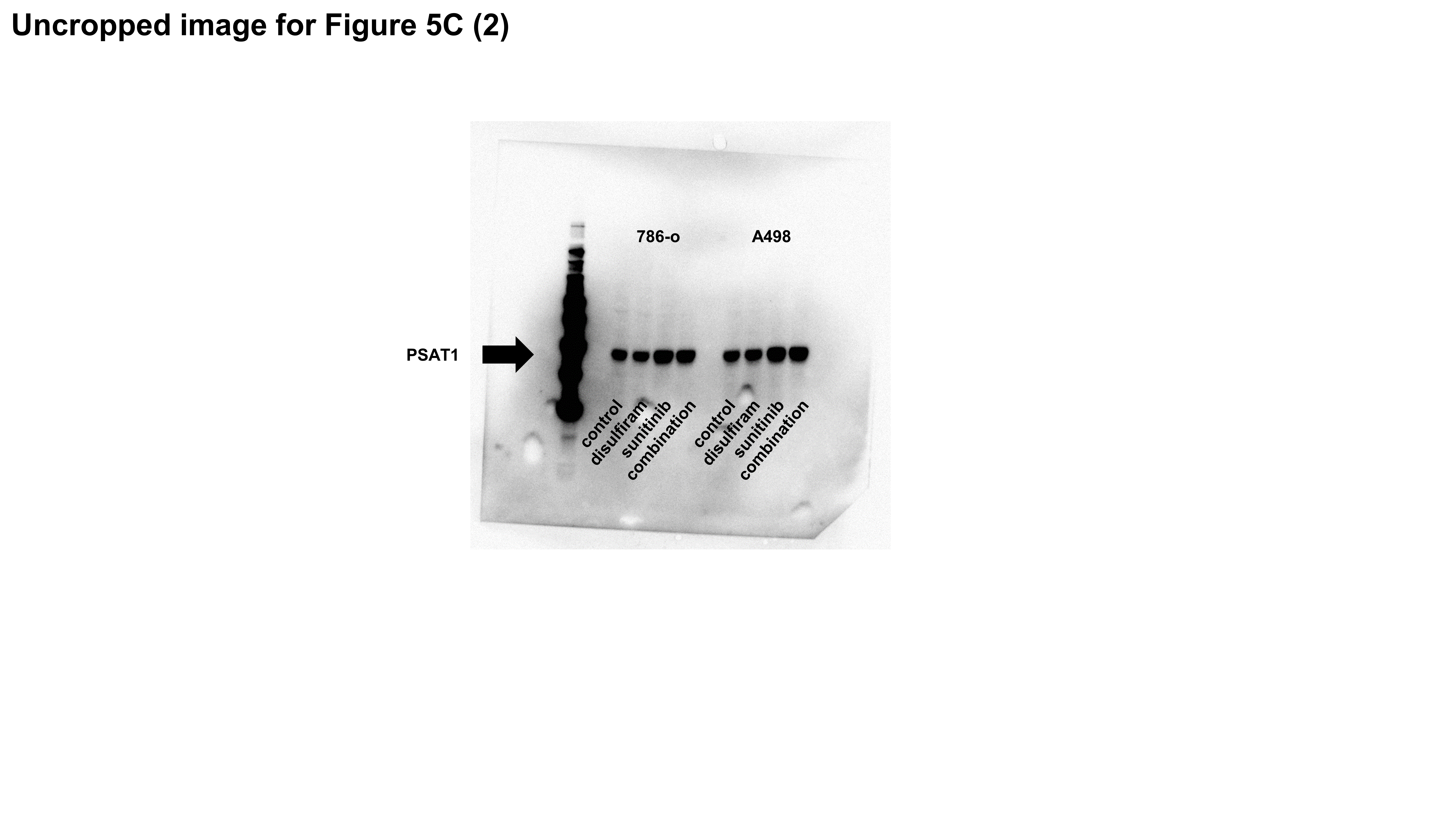

Supplement: S1 File — (ZIP) [file pone.0236119.s003.zip › S8 Fig..TIF]

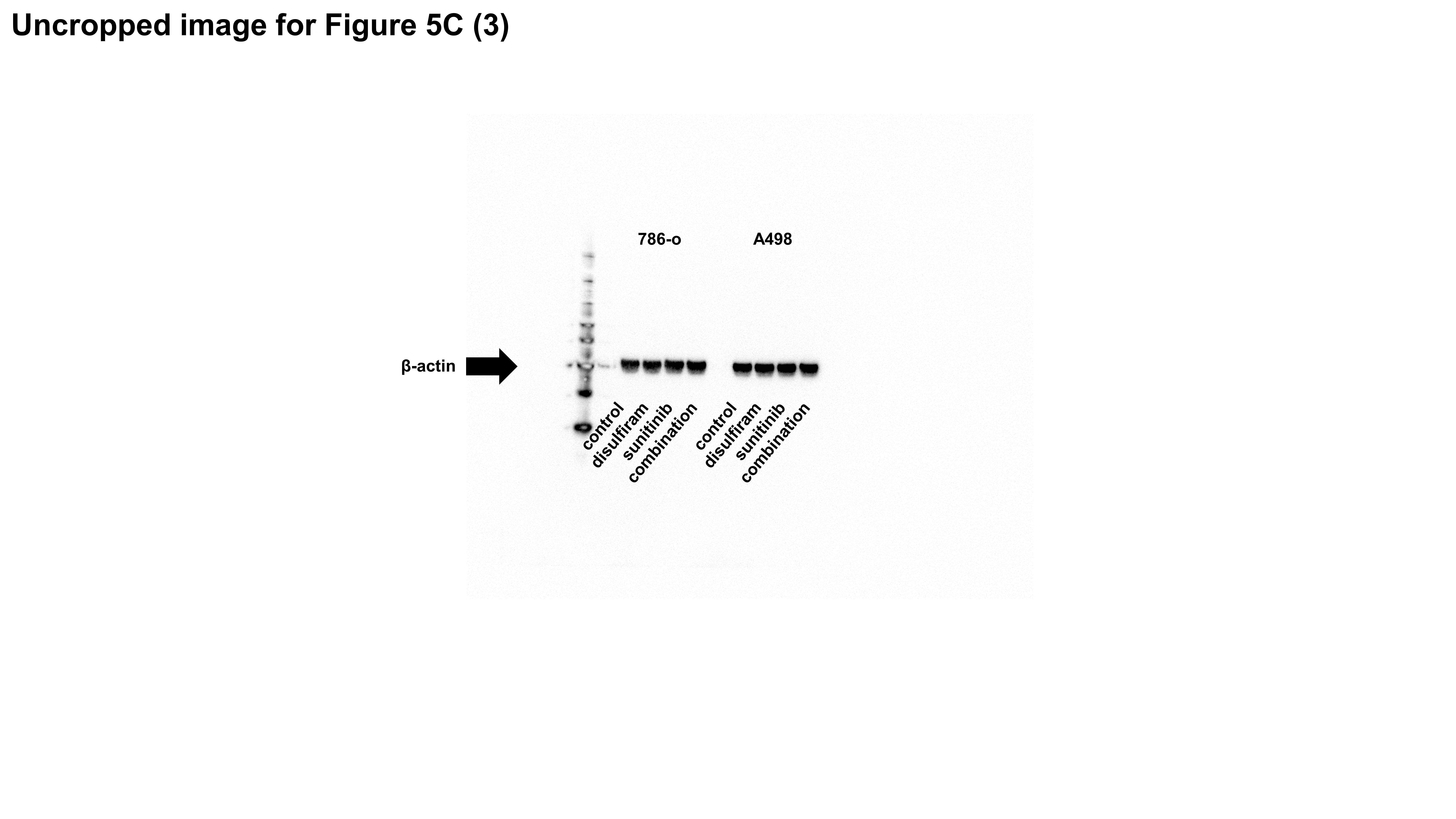

Supplement: S1 File — (ZIP) [file pone.0236119.s003.zip › S9 Fig..TIF]
